# GenerRNA: A generative pre-trained language model for *de novo* RNA design

**DOI:** 10.1371/journal.pone.0310814

**Published:** 2024-10-01

**Authors:** Yichong Zhao, Kenta Oono, Hiroki Takizawa, Masaaki Kotera

**Affiliations:** 1 The University of Tokyo, Tokyo, Japan; 2 Preferred Networks, Inc., Tokyo, Japan; Abu Dhabi University, UNITED ARAB EMIRATES

## Abstract

The design of RNA plays a crucial role in developing RNA vaccines, nucleic acid therapeutics, and innovative biotechnological tools. However, existing techniques frequently lack versatility across various tasks and are dependent on pre-defined secondary structure or other prior knowledge. To address these limitations, we introduce GenerRNA, a Transformer-based model inspired by the success of large language models (LLMs) in protein and molecule generation. GenerRNA is pre-trained on large-scale RNA sequences and capable of generating novel RNA sequences with stable secondary structures, while ensuring distinctiveness from existing sequences, thereby expanding our exploration of the RNA space. Moreover, GenerRNA can be fine-tuned on smaller, specialized datasets for specific subtasks, enabling the generation of RNAs with desired functionalities or properties without requiring any prior knowledge input. As a demonstration, we fine-tuned GenerRNA and successfully generated novel RNA sequences exhibiting high affinity for target proteins. Our work is the first application of a generative language model to RNA generation, presenting an innovative approach to RNA design.

## 1 Introduction

RNAs(Ribonucleic acids) play essential roles in broad biological phenomena. The design and engineering of RNA is a promising tool in the progress of advanced therapeutics and biotechnology. For example, RNA aptamers, which can specifically target proteins or small molecules, have been engineered and utilized in gene silencing therapies through targeted siRNA delivery and as alternatives to fluorescent proteins in diagnostic techniques [1, 2]. Furthermore, RNA aptamers have been developed as therapeutic agents, as demonstrated by the FDA-approved drug Macugen^®^ (pegaptanib) for the treatment of age-related macular degeneration. Another example is the design of 5’ untranslated regions (UTRs) in mRNA, which can impact translation efficiency and is particularly valuable in the development of mRNA vaccines [3].

Historically, RNA design methodologies have been grounded in empirical approaches and directed evolution in wet-lab settings, which is a process plagued by high costs and low efficiency [4]. Subsequently, the introduction of computational techniques permitted the exploration of RNA sequences with specific secondary structures, which further advanced RNA design [5]. However, the reliance on pre-defined RNA structural configuration and other a priori knowledge has imposed constraints on the adaptability of these methods [6]. To address this, there have been recent efforts to integrate deep learning approaches into RNA generation, such as RNAGEN [7], which utilizes WGAN-GP [8], a variant of the generative adversarial network (GAN) [9] architecture, to generate new sequences with similar characteristics to training sequences. Moreover, it can leverage external prediction models to guide the model in further optimization for target features. This approach has enabled the successful generation of PIWI-interacting RNA(piRNA) sequences that specifically bind to target proteins. However, GAN-based models are generally considered less suitable for generating long sequences due to significant challenges in maintaining contextual coherence and ensuring sequence diversity [10, 11], which may limit the applicability of RNAGEN to other RNA generation tasks. RfamGen [12] is another noteworthy contribution in this field. It utilizes covariance model (CM) [13], which is a statistical framework that incorporates alignment and consensus secondary structure within RNA family data, in combination with variational autoencoders (VAEs) [14]to successfully generate functional sequences across diverse RNA families. This approach has efficiently produced several functional RNAs. While RfamGen overcomes the dependency on RNA inverse folding, it still operates within the context of RNA families, relying on constraints such as multiple sequence alignment (MSA) and consensus secondary structure.

The recent advancements in large language models have opened new possibilities for RNA sequence generation. These models learn from massive unlabeled datasets, produce meaningful representations, and generate high-quality text [15]. This success has resonated in the biological and chemical domains [16–21], offering a new paradigm where the structure and function of biomolecules can be handled within the framework of a natural language model. For instance, ProGen, a large-scale protein language model, generates protein sequences with predictable functions across diverse protein families, also aiding in the exploration of protein space [22]. Similarly, in the realm of small molecule design, there has been a proliferation of published generative language models utilizing string-based molecule representations, garnering considerable attention [23]. As for RNA research, pre-trained language models like RNA-FM, UNI-RNA, and RNABERT have demonstrated their effectiveness in predicting RNA function and structure [24–26]. However, these language models were specifically trained to capture patterns in RNA sequences rather than generation.

Building on this, we introduce GenerRNA, the first generative language model for RNA. The motivation behind our study is to provide a platform that both eliminates the dependency on external knowledge and offers versatile functionality, as well as to explore the potential of applying LLMs that have been proven successful in protein and small molecule design to RNA. Our model is based on the Transformer decoder architecture [27], which is typically pre-trained on large text datasets to learn underlying syntactic and linguistic patterns in an unsupervised manner. This process entails predicting subsequent words or characters in a text sequence without the need for labels or annotations. GenerRNA was pre-trained on approximately 16 million RNA sequences encompassing 11.6 billion nucleotides, in order to gain a comprehensive understanding of RNA representations across different families, facilitating *de novo* sequence generation. We evaluated the generated RNA sequences from the following standpoints: secondary structural stability measured by minimum free energy (MFE) and novelty assessment by conducting homology searches. Additionally, the nucleotide propensities of the generated sequences are similar to those found in natural sequences. The results reveal that GenerRNA can generate RNA sequences that are novel, nature-like, and structurally meaningful. Furthermore, we fine-tuned the model to generate RNAs with the capability to bind to specific proteins as a demonstration for versatility. *In silico* evaluations indicated that these generated RNAs exhibit high affinity scores with target proteins. Additionally, ablation experiments demonstrated the substantial role of pre-training, allowing for the generation of more rational and novel sequences.

GenerRNA presents a new option for automated RNA design, and our work demonstrates that generative language models have the potential to become a promising approach in RNA engineering.

## 2 Methods

### 2.1 Learning the RNA language

GenerRNA is a language model that processes RNA sequences through a linguistic perspective. By employing unsupervised learning on a large-scale RNA dataset, it is possible to discern the inherent syntax, grammar, and semantics in RNA sequences, thereby mastering the ability to generate the RNA “language”—signifying a capacity to generate RNA sequences akin to nature.

Within this framework, GenerRNA computes the probability for each specific token *x*_*i*_ in a sequence, where a *token* refers to a discrete unit consisting of one or more nucleotides, and the index *i* denotes the token’s position in the sequence. This probability is influenced by the token’s context, determined as the joint probability of preceding tokens in the sequence ranging from *x*_1_ to *x*_*i*−1_. The aggregate probability of a sequence *X* consisting of *L* tokens is derived from the joint probability distribution of its constituent tokens.

$$\begin{array}{c} p\left(X\right)=\prod_{i=1}^{L}p\left(x_{i}|x_{<i}\right) \end{array}$$ (1)

Focusing on sequence generation, GenerRNA employs an autoregressive approach, implying that the model operates in a sequential, left-to-right fashion to construct the sequence one token at a time. This process necessitates that the prediction of each subsequent token depends on all previously generated tokens, ensuring a contextually coherent progression in the sequence generation.

**Fig 1:**
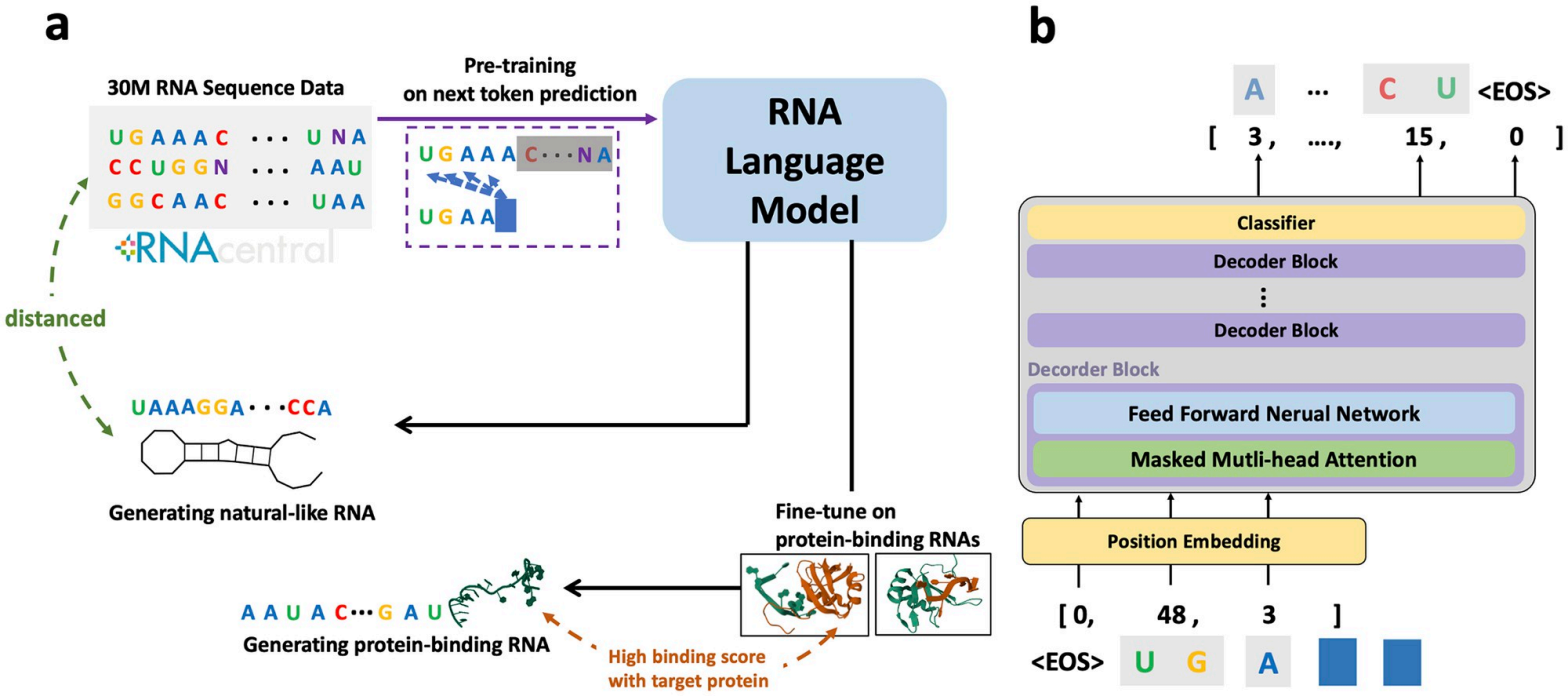
Overview of GenerRNA workflow and design architecture. **a**. GenerRNA undergoes unsupervised pre-training on a large-scale RNA corpus, enabling *de novo* sequence generation. The model can be further fine-tuned to perform specific downstream tasks. **b**. GenerRNA is composed of 24 Transformer decoder layers. The model operates in an autoregressive manner to predict the subsequent token. Both the input and output of the model are in the form of tokens, which are encoded and decoded by a trained tokenizer. A special token (EOS) is used to delimit sequences, indicating the start and end of each sequence. The 3D structures in the diagram were sourced from RNAComposer [28] and the Protein Data Bank [29–31].

Our model is built on the Transformer decoder architecture, which features a series of stacked layers designed to learn the global context within sequences, with each layer incorporating a self-attention mechanism, as illustrated in Fig 1b. This self-attention mechanism within each layer is responsible for interpreting pairwise interactions among all positions in its input sequence. Our decoder-only architecture model consists of 350 million parameters across 24 transformer layers and has a model dimension of 1280. This configuration mirrors the GPT-2-medium model architecture proposed by OpenAI [32].

We trained the model by minimizing the negative log-likelihood (NLL) across the entire dataset. This approach involves the model learning the relationship of each token in a sequence with the preceding tokens.

$$\begin{array}{c} NLL_{\text{seq}}=-\sum_{i=1}^{L}\;\text{log}\;p_{\theta }\left(x_{i}|x_{<i}\right) \end{array}$$ (2)

Here, log *p*_*θ*_(*x*_*i*_|*x*_<*i*_) is the log is the log probability of the *i*th token *x*_*i*_, given the model parameters *θ* and the the preceding context.

GenerRNA was trained for 12 epochs on 11GB of sequence data (S1 Fig in S1 File). The parameter weights were initialized prior to training, with the linear layers initialized using Kaiming initialization [33], while other layers were initialized to a Gaussian distribution with a mean of 0 and a standard deviation of 0.02. The optimizer used was Adam (*β*_1_ = 0.9, *β*_2_ = 0.999) [34] with the learning rate experiencing a warm-up over the first 0.67 epoch to 1e-3 with a linear decay to 1e-4 for the remainder of the training. The training was executed on 16 A100 GPUs and was completed within four days.

### 2.2 Pre-training data preparation

For pre-training, we obtained a total of 34.39 million sequences from RNAcentral (release 22), a comprehensive database that combines RNA sequences from 51 expert databases [35]. The training data comprised over 2,600 Rfam families and 30 types of RNA types such as rRNA, tRNA, and lncRNA, but notably excluded mRNA [36]. In our preprocessing steps, we replaced ‘U’ with ‘T’ while retaining all ambiguity code, and employed MMseqs2 [37] for deduplication with an identity threshold of 80%. The deduplication process improved the generalizability of the validation set and increased the information density of the training data, allowing the model to learn a more diverse range of sequences at the same computational cost.

We then selected sequences shorter than 1,024 tokens once after tokenization, refining the dataset to 16.09 million sequences, encompassing 17.4 billion nucleotides. Of these sequences, 99% were allotted for training, with the remaining 1% allotted to the validation set. Training GenerRNA on longer sequences enables it to learn more complex patterns, but it also significantly increases computational demands. This is due to the quadratic growth in computational complexity with sequence length in the attention mechanism, along with the increased memory required for storing intermediate results [38]. Moreover, current computational biology methods struggle to model excessively long RNA sequences, such as in secondary structure prediction, where there is a challenging trade-off between computational time and accuracy. Given these challenges, we filtered out excessively long sequences from the training data. GenerRNA has a maximum output length and context window of 1024 tokens, corresponding to approximately 4000 nucleotides (nt), which meets the requirements for sequence length in most current RNA design tasks (S3 Fig in S1 File). Additionally, we have released a historical version of the model in our code repository, which supports a maximum output length and context window of 256 tokens, requiring fewer computational resources.

### 2.3 Training tokenizers

Neural networks are inherently incapable of directly manipulating character-based data, necessitating the conversion of sequences into a tokenized format. In the pre-training phase of our model, we utilize Byte-Pair Encoding (BPE) for tokenization [39]. Originally devised as a text compression algorithm, BPE facilitates the representation of variable-length subwords within a fixed vocabulary size. Such capability is notably absent in the *k*-mer tokenization, which is commonly employed in biological sequence analysis. Additionally, compared to the one-hot tokenization, where each base corresponds to one token, the BPE tokenizer compresses the information content, enabling the learning and generation of longer sequences. The straightforwardness and effectiveness of BPE tokenization have garnered widespread popularity in the realm of Large Language Models [40].

To train our tokenizer, we constructed a dataset comprising 1 million sequences randomly extracted from RNAcentral. Throughout this procedure, we experimented with various vocabulary sizes ranging from 512 to 50,296 and observed that larger vocabulary sizes led to single tokens encompassing more nucleotides (S2 Fig in S1 File). We opted for a vocabulary size of 1024 primarily for the following reasons: A larger vocabulary size would significantly reduce the number of tokens in the training data, making it fall below the number we estimated to be suitable for our computational resources based on the Chinchilla Scaling Law [41]. Conversely, a smaller vocabulary size would limit the tokenizer’s ability to compress information, restricting the length of sequences the model could process within the same context window.

### 2.4 Statistical examination of nucleotide propensities

Our research aimed to generate RNA sequences that share similar properties with natural sequences. To realize this, we examined nucleotide distributions under various sampling strategies and parameters. We focused our analysis on three main sampling strategies: greedy search, beam search, and random sampling.

Greedy search persistently chooses the nucleotide with the highest probability, while beam search retains and explores a set of highly probable token sequences within a predefined beam width. We evaluated beam sizes ranging from 5 to 100, incrementing by 5. Due to the inherent determinism of outputs generated by greedy and beam searches, we generated sequence prefixes by creating all possible combinations of A, U, G, and C in the first five nucleotide positions. This approach allowed us to generate 1,024 sequences for each parameter configuration in both search methods.

Random sampling chooses from the top *k* tokens according to the next token’s probability distribution. For this method, we set the temperature at 1.0, a parameter that modulates generation randomness by modifying the logits before the softmax function. We varied the *top*_*k*_ value, ranging from 2 to 1000, creating 1000 sequences for each configuration. We calculated and compared the distribution of *k*-mer frequencies between the generated and natural sequences for different *k*-mer sizes, with *k*-values ranging from 1 to 6. Using a sliding window of 1 bp, we accommodated a total of 4^k^ unique *k*-mers for each value of *k*. Finally, to quantify the differences in *k*-mer frequency between the generated and natural sequences, we employed the Kullback-Leibler (KL) Divergence:

$$KLD=\sum_{i=1}^{64}P\left(i\right)\;\text{log}\;(\frac{P(i)}{Q(i)})$$

Here, *P*(*i*) represents the frequency of the *i*-th *k*-mer in the generated sequences, whilst *Q*(*i*) denotes its frequency in natural sequences. This formula sums over all 4^*k*^ possible *k*-mers(such as AAA, AAU, AAG, etc. when *k* = 3).

### 2.5 Evaluation of generated RNAs

We investigated the minimum free energy (MFE) of generated RNA sequences as an indicator of secondary structural stability through a control experiment. Since longer RNA sequences tend to have more opportunities for intramolecular interactions, which can result in lower MFE values [42], we controlled for sequence length by matching the length distribution across all experimental groups. First, we selected 2,000 sequences generated by our model to form the **Generated Group**. We then randomly sampled 2,000 natural sequences with the same length distribution as the generated sequences, creating the **Natural Group**. For each sequence in the Generated Group, we also generated a corresponding sequence of the same length composed of random AUGC nucleotides, which we designated as the **Random Group**. To further control for the influence of nucleotide composition (e.g., GC content, *k*-mer distribution), we created a **Shuffled Group** by shuffling the nucleotide sequences from the Generated Group using *uShuffle* [43], which allowed us to shuffle the sequences while maintaining their 3-mer distribution.

Additionally, to evaluate the robustness of our model’s performance across different sequence lengths, we calculated the MFE distribution for sequences of varying lengths: we sampled 50 generated sequences for each length interval respectively, ranging from 0 to 4,000 nucleotides in 50-nucleotide increments, and prepared a corresponding Shuffled Group for each interval. All MFE calculations were performed using RNAFold [44].

To evaluate the similarity between the generated RNA and known RNA sequences, we employed the sequence search API provided by RNAcentral, which operates on nhmmer [45]. The nhmmer, based on hidden Markov models, demonstrates enhanced sensitivity in detecting sequence similarities when compared to BLAST [46]. Furthermore, the sequence search API of RNAcentral offers improved efficiency through parallel computing and target database splitting, enabling faster search times than when running nhmmer on a local environment [47]. Moreover, this API allows us to simultaneously search Rfam [48] to map query sequences to RNA families. We conducted searches with 1,000 of our generated sequences as queries against the comprehensive sequence database in RNAcentral (S1 Table in S1 File).

### 2.6 Fine-tuning GenerRNA on protein-binding RNAs

**Fig 2:**
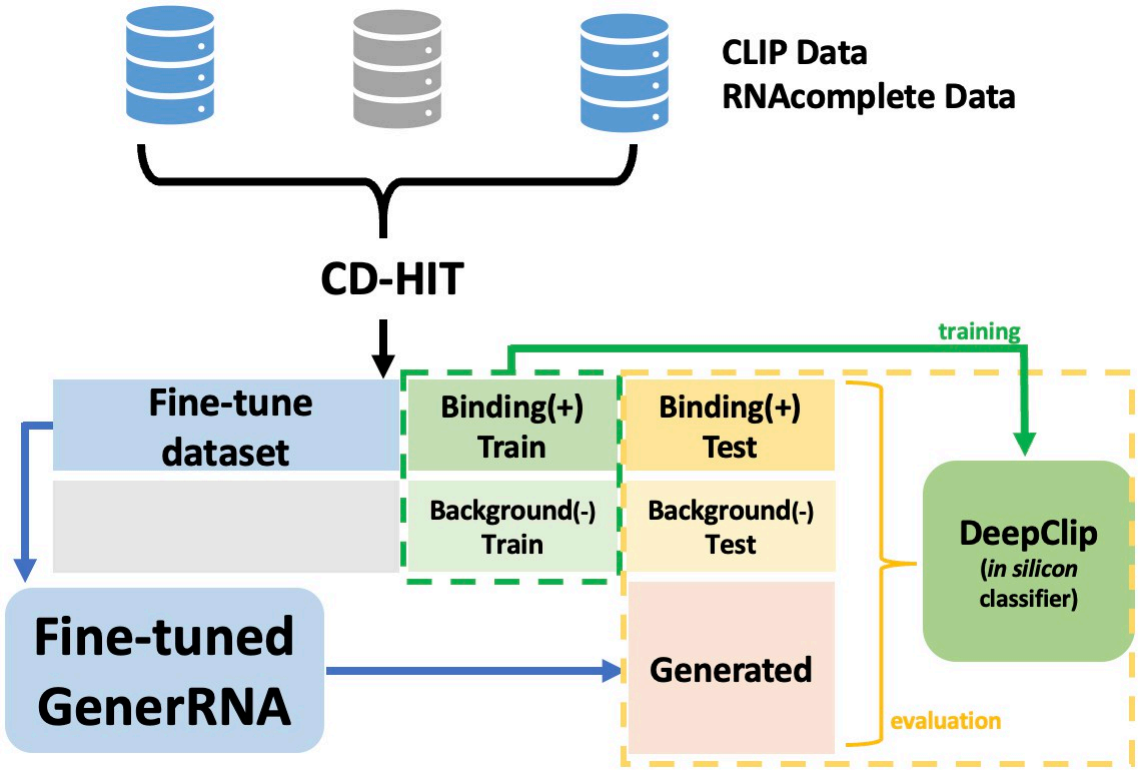
Schematic representation of fine-tuning on protein-binding RNA: The experimental data collected were consolidated and deduplicated using CD-HIT to prevent sequence overlap and data leakage across different datasets. The data were divided into three parts: one for training DeepClip, another for the test set, and the last for fine-tuning the GenerRNA dataset. The trained DeepClip model was subsequently utilized to score sequences generated by GenerRNA.

With the goal of generating novel RNA sequences that bind to specific proteins, we fine-tuned GenerRNA (Fig 2). We then evaluated the binding affinity between artificially generated RNAs and the target proteins by employing DeepClip [49], an *in silico* method leveraging deep learning techniques to model protein-RNA interactions.

Four *in vivo* CLIP datasets [50–52] and five *in vitro* RNAcompete datasets [53] were collected for the ELAVL1 protein. ELAVL1 has been implicated in a variety of biological processes and has been linked to a number of diseases, including cancer [54]. The positive sequences (protein-binding sequences) and negative sequences (background sequences sampled from non-target regions) were grouped independently. To minimize the risk of data leakage due to sequence overlap between training and testing sets, we applied a CD-HIT [55] identity threshold of 80% for deduplication. This process yielded 78,085 positive and 101,178 negative sequences. Similarly, for the SRSF1, which acts as an oncoprotein and an important target for cancer therapy [56], we obtained 128,053 positive and 107,950 negative deduplicated sequences from three CLIP datasets [57–60] and five RNAcompete datasets [53, 61].

Regarding each protein’s binding RNA, 60% of the sequences were allocated for the fine-tuning of GenerRNA, while 30% were used as positive data for training DeepClip, respectively. The remaining 10% was set aside for testing. Subsequently, an equivalent count of sequences from the negative set was randomly selected to function as the negative training, validation, and testing set for DeepClip. We fine-tuned GenerRNA using the ELAVL1 and SRSF1 Clip datasets for 500 epochs each. The learning parameters were consistent with those of the pre-training phase, except for a learning rate of 1e-4 without any warmup or scheduler. Throughout the fine-tuning process, both the validation loss and perplexity exhibited a consistent reduction during the training period.

### 2.7 Evaluation of the generated protein-binding RNAs

We employed a fine-tuned version of GenerRNA to generate protein-binding RNAs *de novo*. The decoding parameters used during sequence generation were consistent with those applied in our pre-tuning experiments. The number of sequences generated was equivalent to those in our curated test set.

We trained DeepClip over ten epochs, selecting the checkpoint that demonstrated the best performance on the validation set. We then computed binding affinity scores for three distinct sequence datasets: **1. Dataset Generated by GenerRNA**, **2. Positive (binding) testset**, and **3. Negative (background) testset**. These affinity scores ranged from a minimum of 0 up to a maximum of 1.0, where a higher score indicates a stronger binding affinity. It should be emphasized that for each specific target protein, the fine-tuning and evaluation of GenerRNA were conducted independently, ensuring tailored optimization for each protein-binding RNA.

In addition to evaluating the affinity of the generated protein-binding RNA, we also conducted a homology search between the generated sequences and existing sequences using nhmmer. We designated the curated protein-binding RNA dataset as a database, with all generated RNA specific to that protein serving as the query. We intentionally set a relatively relaxed threshold to enhance the sensitivity for alignments (S1 Table in S1 File).

Furthermore, we attempted to evaluate similar existing RNA generation models for comparison. However, it was not feasible to construct a truly meaningful multiple sequence alignment and covariance model (CM) based on our curated dataset, which prevented us from applying RfamGEN to this task. On the other hand, RNAGEN is capable of incorporating other constraint models to optimize target features and also supports direct training on RNA sequence datasets without relying on external knowledge. Therefore, we trained RNAGEN on the same dataset as GenerRNA, subsequently evaluating the generated sequences using the same methods.

### 2.8 Ablation study

Typically, large-scale model pre-training enhances a model’s generalization performance and robustness. It enables us to fine-tune the model with fewer computational resources and reduced data, facilitating the transfer of acquired knowledge to specific subtasks.

Here, we conducted an ablation study to assess the impact of large-scale pre-training in the context of RNA sequence generation. We trained a model from scratch on protein-binding RNAs, intentionally bypassing large-scale pre-training, to serve as a control in the ablation group. The model architecture employed was consistent with that used in previous experiments. However, due to the relatively small amount of training data compared to the number of model parameters, rapid overfitting was anticipated. We adjusted the learning hyperparameters and implemented an early stopping strategy to mitigate overfitting. Subsequently, we utilized the ablation model and the fine-tuned GenerRNA to independently generate 1000 sequences each. These sequences were then subjected to homology searches using nhmmer, targeting a database containing RNA sequences that bind to the target protein. The parameter settings for nhmmer were identical to those introduced in the previous section.

## 3 Results

### 3.1 Sampling strategies

Various natural language processing tasks exhibit a propensity for distinct sampling strategies. By measuring the KL divergence in nucleotide distribution between generated and natural sequences, we identified the most suitable sampling strategies and parameters for generating natural-like RNAs.

The greedy search method opts for the token of the highest probability, leading to consistent results. This approach is particularly effective for structured QA tasks but often results in repetitive segments during long text generation [62]. Notably, a similar phenomenon has also been observed in the generation of RNA sequences. Beam search aims to mitigate this issue by retaining a set of most probable token sequences, proving to be beneficial in tasks needing a balance between efficiency and quality like machine translation [63, 64]. Our results indicate that while RNA sequences generated using beam search tend to exhibit fewer repetitive segments relative to those generated via greedy search, a significant disparity remains in the distribution of *k*-mers between the generated sequences and natural RNA sequences.

**Fig 3:**
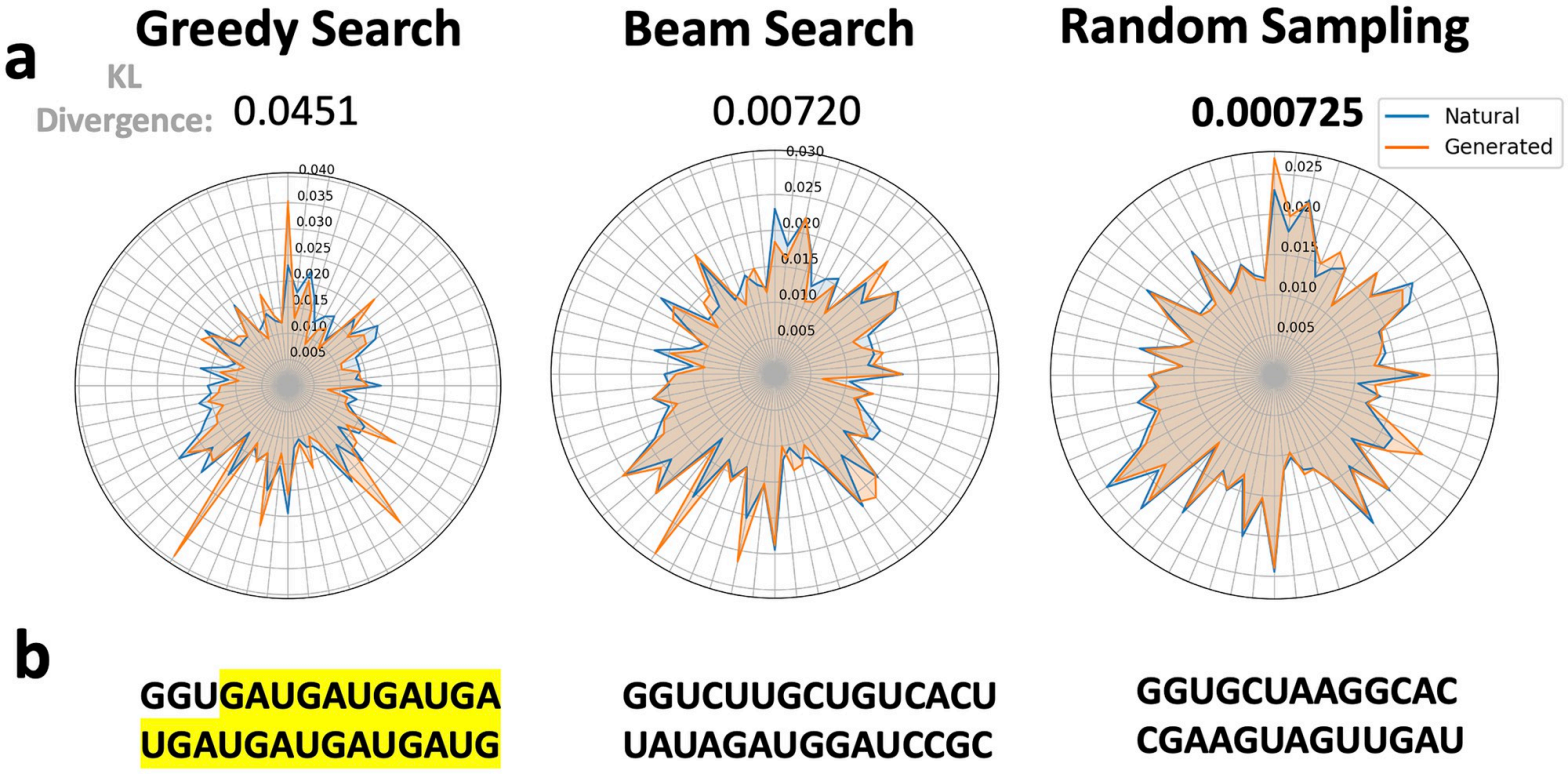
**a**. Radar charts depicting the 3-mer nucleotide distribution of sequences generated under different sampling strategies, where each axis of the radar chart represents a specific 3-mer (e.g., AAA, AAU, etc.). The charts are accompanied by the KL divergence, which compares these distributions to those of natural sequences. **b**. Examples of generated sequences. **a. b**. Greedy search tends to generate RNA with repetitive fragments, as highlighted, and its 3-mer distribution significantly differs from that of natural sequences (KL divergence = 0.0959). Beam search partially alleviates this issue. However, the nucleotide distribution in its generated RNA still deviates from that observed in natural sequences (KL divergence = 0.0115). The nucleotide distribution in sequences under random sampling closely aligns with that of natural sequences (KL divergence = 0.00134).

Random sampling, which selects from the top *k* tokens based on the next token’s probability distribution, has been found to produce outputs with greater randomness and a more natural feel in long text generation [65]. Our statistical evaluations reveal that RNA sequences generated using this method exhibit a *k*-mer distribution that most closely resembles natural sequences among all assessed sampling strategies (Fig 3). Setting the *top*_*k*_ value to around 250 typically yields the smallest KL divergence across various *k*-mers when compared to natural sequences (S4 Fig in S1 File).

### 3.2 Minimum free energy of generated RNAs are similar to that of natural ones

The minimum free energy of an RNA sequence is defined as the energy of its secondary structure that contributes the least to the free energy, with a lower MFE that signifies a more stable RNA secondary structure. To eliminate the impact of sequence length on MFE, we ensured a consistent sequence length distribution across all control groups. Additionally, nucleotide distribution in the Shuffled Group was aligned with that of the Generated Group, thereby controlling for factors such as GC content that could affect MFE.

**Fig 4:**
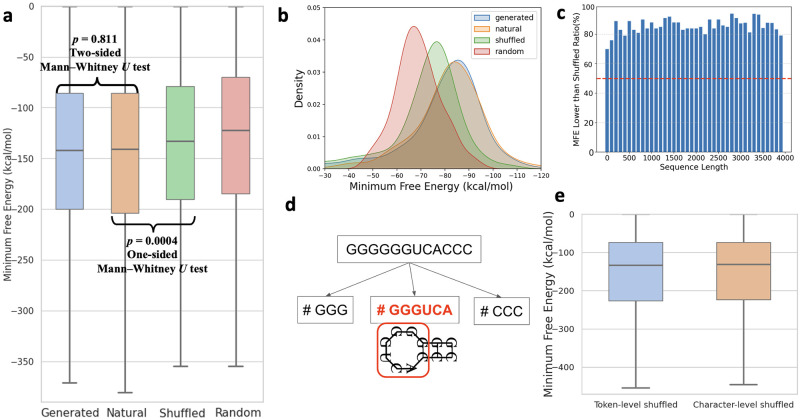
Statistical analysis of generated sequences. **a**. This plot displays the distribution of minimum free energy among four groups: Natural Group, Generated Group, Shuffled Group, and Random Group. There is no significant difference in the MFE distribution between generated and natural sequences, whereas the MFE of generated sequences is significantly lower than that of shuffled and random sequences. **b**. MFE distribution among different collections with sequences length set at 250 nt. **c**. Proportion of generated sequences that exhibit a lower MFE compared to their respective shuffled sequences at diverse length intervals. The proportion across all intervals is greater than 0.5, indicating that GenerRNA is able to generate sequences with lower MFE across a variety of length ranges. **d**. The BPE tokenizer, which learns common nucleotide patterns in RNA, might also unintentionally capture motifs that affect the stability of secondary structures. **e**. When the token sequences generated by the model are shuffled prior to decoding into nucleotide sequences, the MFE distribution of these sequences showed no statistically significant difference compared to the sequences shuffled at the character level. This indicates that the doubt raised in **d** does not hold, or at least does not have a significant impact.

Results indicate that the mean MFE of the generated sequences was slightly higher than that of the natural sequences (-174.7 vs –177.9 kcal/mol), while there was no statistically significant difference between two groups (two-sided Mann-Whitney *U* test, p-value = 0.811). Additionally, the MFE of the generated sequences was significantly lower than that of both the shuffled and random groups, as determined by a one-tailed Mann-Whitney *U* test (p-value = 4.43e-3 and 6.65e-11). Besides, Fig 4c. depicts that the majority of the generated sequences, regardless of their length, had a lower MFE than its shuffled sequences. These findings indicate that our model can generate sequences with relatively stable secondary structures across a broad range of sequence lengths.

Potential concerns arise given that the Byte Pair Encoding (BPE) tokenizer was trained on a dataset of one million sequences, possibly leading it to learn common nucleotide arrangements and merge these nucleotide combinations into individual tokens. This could inadvertently result in the learning of RNA motifs, which are sequence and secondary structure-related feature patterns in RNA [66]. To investigate whether this was a contributing factor to the observed lower MFEs, we conducted an experiment where the token sequences output by the model were shuffled prior to being decoded back into nucleotide sequences. In this approach, while the nucleotide combinations within the same token stayed adjacent post-decoding, the overall global context was entirely disrupted. We generated 1,000 sequences in this manner, designated as the Token-level-shuffled Group. For comparison, we further shuffled these sequences completely, maintaining the length distribution and base composition unchanged, and denoted these as the Character-level-shuffled Group. As shown in Fig 4e., there was no statistically significant difference in the MFE distribution between these two groups (two-sided Mann-Whitney *U* test, p-value = 0.811). This outcome dispels our initial concerns and substantiates that the lower MFEs of the generated sequences are primarily attributed to the contextual information learned by GenerRNA rather than the BPE tokenizer having learned inherent nucleotide patterns.

### 3.3 Generated RNAs are distanced from existing sequences

To assess the novelty of the sequences generated, a homology search was conducted against a comprehensive database of known sequences. The metric employed for this evaluation was identity, defined as the proportion of matching positions in the aligned subsequences. Additionally, an e-value of 0.1 was set as the threshold for determining the reliability of the alignments. The e-value threshold of 0.1 is higher than that commonly used in sequence searches, indicating more sensitive searching and more hits.

$$\text{Identity}=(\frac{n_{\text{identical}}}{n_{\text{align}}})\times 100\%$$ (3)

**Fig 5:**
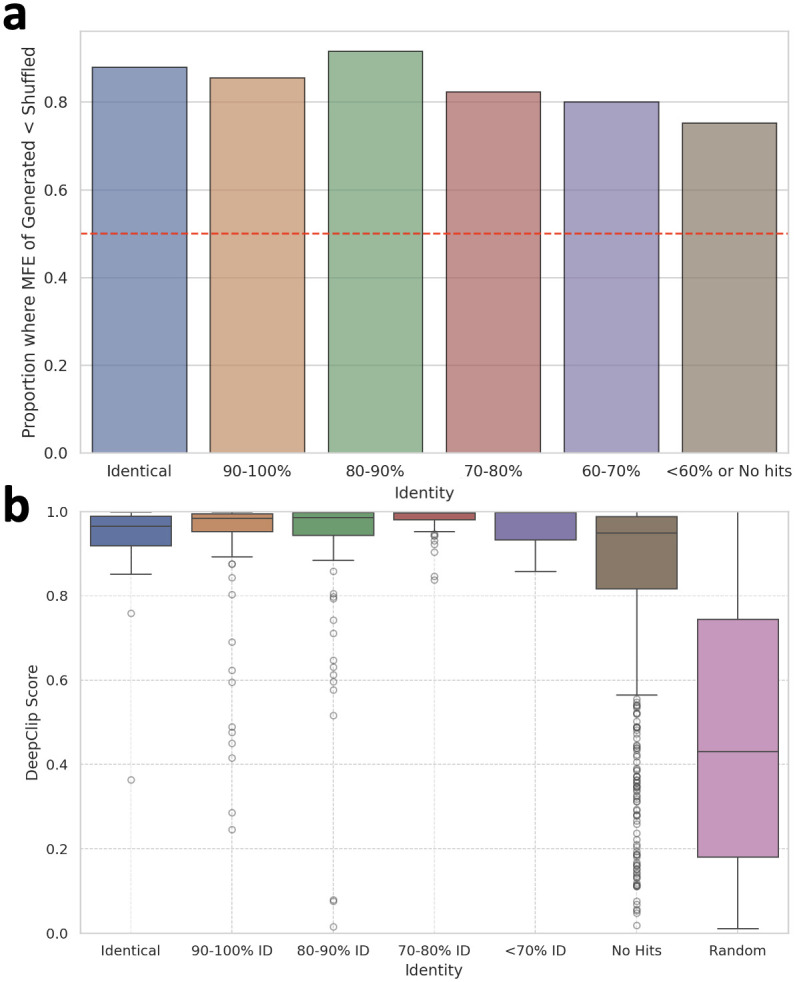
**a**. The ratio of sequences exhibiting lower MFE compared to their corresponding shuffled sequences at diverse identity levels. **b**. Distribution of affinity scores with the target protein (ELAVL1) at varying identity levels and with a randomly shuffled group (Results for SRSF1 are shown in S6 Fig in S1 File). **a.&b**. It is observed that numerous instances of RNA with stable secondary structures or high affinity scores can be found even among sequences with an identity of less than 90% or those that are non-hits.

Our findings revealed that 3.9% of the analyzed sequences exhibited 100% identity with those in the database, with an e-value less than 0.1, signifying a perfect match of all nucleotides in the aligned subsequences for these cases. Nonetheless, another 69.9% of the generated sequences aligned with known sequences (The identity distribution of those sequences can be found in S5 Fig in S1 File), meanwhile the aligned subsequences were not identical, and 26.2% of the sequences did not align significantly with any known sequences at an e-value threshold of 0.1. Also, 70.6% of the generated sequences could be assigned to an existing RNA family. As demonstrated in Fig 5a., in cases where sequences did not show substantial alignments or had less than 90% identity, the majority still exhibited lower compared to their shuffled ones. This suggests that GenerRNA generates novel RNA sequences that maintain a significant level of secondary structure, even when diverging from known sequences.

### 3.4 Fine-tuned GenerRNA generates protein-binding RNAs

In natural language processing, fine-tuning a pre-trained model on specific tasks boosts task-specific performance or generates articles in a particular style while retaining a general understanding of language syntax, grammar, and semantics. Such a procedure necessitates relatively fewer data and computational resources. In our study, we fine-tuned the GenerRNA using RNA data binding to specific proteins, and estimated the affinity scores between the generated sequences and target proteins using in silico computational methods.

**Fig 6:**
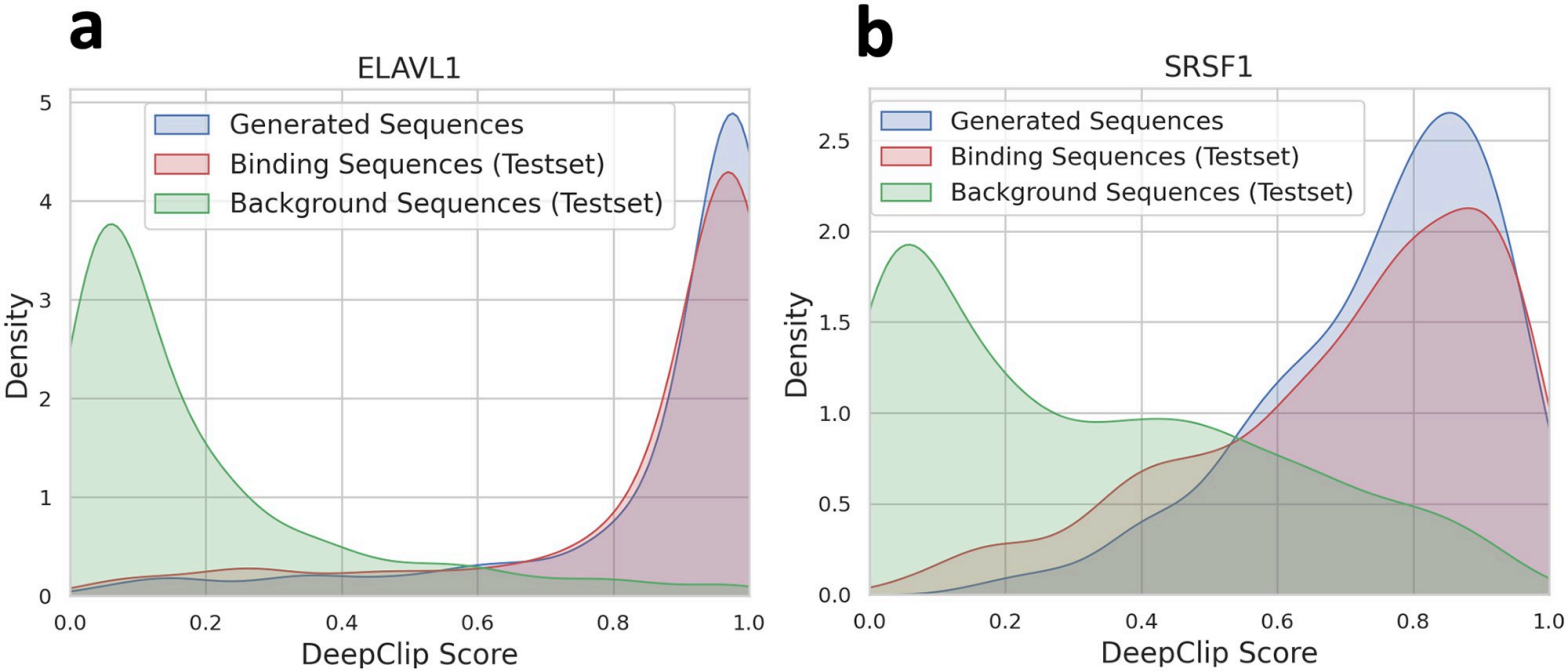
**a.&b**. Illustrate the affinity scores of the sequences generated by GenerRNA, along with the positive and negative test sequences, in relation to their respective target proteins (ELAVL1, SRSF1). The affinity scores of the sequences produced by GenerRNA were significantly higher than those of the negative control group, and were comparable to the positive group.

As depicted in Fig 6, sequences generated by GenerRNA exhibit a pronounced specificity toward the target protein. For both examined proteins, the affinity scores of the generated sequences not only significantly exceed those of the negative test set but also marginally surpass the mean scores observed in the positive test set. Specifically, for ELAVL1, the average affinity score of generated RNAs was 0.872 compared to 0.850 in the positive test set, and for SRSF1, it was 0.720 versus 0.713. Furthermore, homology searches reveal that only a small fraction of the RNA sequences generated by GenerRNA that bind to ELAVL1 and SRSF1 proteins formed identical alignments with binding sequences, with 2.20% for ELAVL1 and 1.30% for SRSF1. Notably, a majority of the sequences, 70.9% for ELAVL1 and 67.2% for SRSF1, were not aligned with any known binding sequences (The identity distribution of aligned sequences can be found in S7 Fig in S1 File). As illustrated in Fig 5b., many instances of RNA with high-affinity scores were observed even among sequences with less than 90% identity or those that did not form alignments. It is important to mention that the tool adopted for our homology analyses is highly sensitive, and we have intentionally set more relaxed thresholds to bolster the detection sensitivity for alignments.

Furthermore, we performed a comparative analysis of RNA sequences generated by RNAGEN (S1 Note in S1 File). On the ELAVL1 dataset, RNAGEN produced sequences that, while more closely resembling the training data, exhibited slightly lower average affinity scores compared to those generated by GenerRNA (0.773 vs 0.872). As for the SRSF1 protein, we were unable to successfully train a model capable of generating effective binding sequences. However, it is important to note that RNAGEN’s complete workflow includes an additional indirect optimization targeting specific proteins. However, we opted not to utilize this approach for two main reasons: first, RNAGEN depends on existing trained DeepBind models to target proteins, but the repository hosting these models has been inaccessible for an extended period; second, optimizing RNAGEN with DeepBind and subsequently evaluating the sequences using similar computational models could undermine the objectivity of our evaluation, as it would not accurately reflect the generative model’s performance under standard conditions. Thus, although our comparison does not fully exploit RNAGEN’s capabilities, it demonstrates that in the absence of external knowledge, GenerRNA’s Transformer-based model more effectively captures the critical features of RNA sequences.

Finally, we conducted an ablation study of the impact exerted by large-scale pre-training on tasks related to generating RNA sequences in downstream applications. The ablation model, which is devoid of pre-training, exhibited a slightly higher validation loss compared to the fine-tuned GenerRNA on the ELAVL1 dataset, recording values of 4.11 and 3.81, respectively. Similarly, the ablation model trained on SRSF1-binding RNAs achieved its optimal validation loss at 4.65, which was marginally higher than the 4.35 achieved by the fine-tuned GenerRNA (S8 and S9 Figs in S1 File). However, despite exhibiting a closer but slightly higher validation loss compared to GenerRNA, the ablation model could only produce sequences characterized by a lack of originality. These sequences predominantly mirrored existing data, indicating a tendency towards mere replicating training data rather than a genuine generation (as demonstrated in Table 1). We speculate that the pre-training on extensive datasets typically empowers the model to internalize a wide range of features and patterns inherent to the RNA data, which offers a robust starting point that enhances the model’s capacity for fine-tuning on specific tasks. This leads to improved generalization and the ability to generate RNA sequences that are not only novel but also adhere to the “grammar” of RNA.

**Table 1:** Comparison of sequence novelty in ablation model and fine-tuned GenerRNA: The fine-tuned GenerRNA generates a greater number of novel RNAs unaligned with known RNAs and fewer sequences identical to known RNAs.

	ELAVL1		SRSF1	
	Identity	No Hits	Identity	No Hits
Ablation Model	90.9	0.11	85.7	1.12
Fine-tuned GenerRNA	**2.20**	**70.9**	**1.30**	**67.2**

## 4 Discussion

### 4.1 Implication

Biological sequences harbor a wealth of information, covering evolutionary history, survival strategies, and even blueprints for future development. AI language models serve as translators for reading and writing this enigmatic language. The ever-increasing number of RNA sequences in public databases, coupled with advancements in natural language model architectures, provides a foundation for constructing models proficient in generating biological sequences.

Our development of GenerRNA marks the first instance of a large-scale. Our model learns RNA from a linguistic perspective to gain the ability to “speak” this language. Compared to existing RNA generation models, our approach offers several advanced features, including the ability to function without relying on predefined secondary structures or any external prior knowledge, the flexibility to be fine-tuned for various tasks and datasets, and the capability to handle longer sequences.

Experimental results show that GenerRNA can generate RNA with secondary structural stability similar to known sequences, while maintaining distinctiveness from them. The generated sequences include many novel yet “RNA-grammar-compliant” sequences, which broaden our sampling of the RNA space. Additionally, to demonstrate that GenerRNA holds promise as a versatile platform for RNA generation, we validated that a fine-tuned GenerRNA can generate RNA sequences with high binding affinity to target proteins, while preserving their uniqueness from existing sequences.

### 4.2 Limitations and future work

GenerRNA represents the initial advancement of large-scale generative language models in the realm of RNA. However, several issues remain to be explored to develop more advanced language models tailored for RNA sequences. For example, GenerRNA utilizes a BPE tokenizer to compress sequences, which helps reduce computational costs, but it may also overlook important chemical interactions that occur between individual nucleotide pairs. Another instance is that mRNA was absent in our pre-training dataset, while the generation of the untranslated region of mRNA is considered a promising direction. Moreover, RNA has yet to experience its “AlphaFold moment” [67, 68]. High-quality datasets and computational methods in RNA research are relatively absent compared to protein realm, meaning there is no golden dataset or a widely accepted benchmark for us to comprehensive compare our models with others, including qualitative or quantitative assessments of RNA secondary and tertiary structures.

Looking ahead, we anticipate that future developments in RNA generative language models will also cover numerous directions yet not be confined to them. One promising avenue is to explore downstream applications of generative RNA language models. These include the generation of functional RNAs, aiding in the design of RNA vaccines, and the development of nucleic acid therapeutics. Another direction involves scaling. Similar advances in protein-related fields have shown that models with larger parameter sizes generate novel protein structures more effectively and may demonstrate emergent abilities in specific tasks [69, 70]. This potential scalability could further utilize the ability of the RNA language model, warranting further investigation. Lastly, controlled generation is also a valuable field in which to develop. Techniques such as prompt tuning [71] could be instrumental in directing these models to generate sequences that belong to specific families or possess desired characteristics.

## Supporting information

S1 File Supplementary information. This file includes additional supplementary, tables, and notes.
(PDF)
