# Supplementary material for: GenerRNA: A generative pre-trained language model for de novo RNA design
Source: PLoS One. 2024 Oct 1;19(10):e0310814. doi: 10.1371/journal.pone.0310814 (PMC11444397; doi:10.1371/journal.pone.0310814)
Supplement: S1 File — This file includes additional supplementary, tables, and notes. (PDF) [file pone.0310814.s001.pdf]

GenerRNA: A generative pre-trained language model for *de novo* RNA design

Supplementary Information

Yichong Zhao, Kenta Oono, Hiroki Takizawa, and Masaaki Kotera

Table of Contents

1. **S1 Fig. Loss during pre-training**
   Page 3
2. **S2 Fig. Effect of Vocabulary Size on Token Length and Ratio of Original/ Tokenized Sequence Length**
   Page 3
3. **S3 Fig. Length distribution of generated sequences and sequences in the training dataset**
   Page 4
4. **S1 Table. Hyperparameters of nhmmer for homology search**
   Page 5
5. **S4 Fig. KL-divergence of *k*-mer distributions between generated sequences under different sampling strategies and natural sequences at various *k*-mer lengths**
   Page 6
6. **S5 Fig. Identity distribution of sequences generated by pre-trained model and aligned to any natural sequences.**
   Page 7
7. **S6 Fig. Distribution of affinity scores with the target protein (SRSF1) at varying identity intervals.**
   Page 8
8. **S7 Fig. Identity distribution of sequences generated by fine-tuned model and aligned to the training data**
   Page 8
9. **S1 Note. Detail about the comparative experiment**
   Page 9
10. **S8 Fig. loss during fine-tuning**
    Page 10
11. **S9 Fig. Loss during ablation experiment**
    Page 10

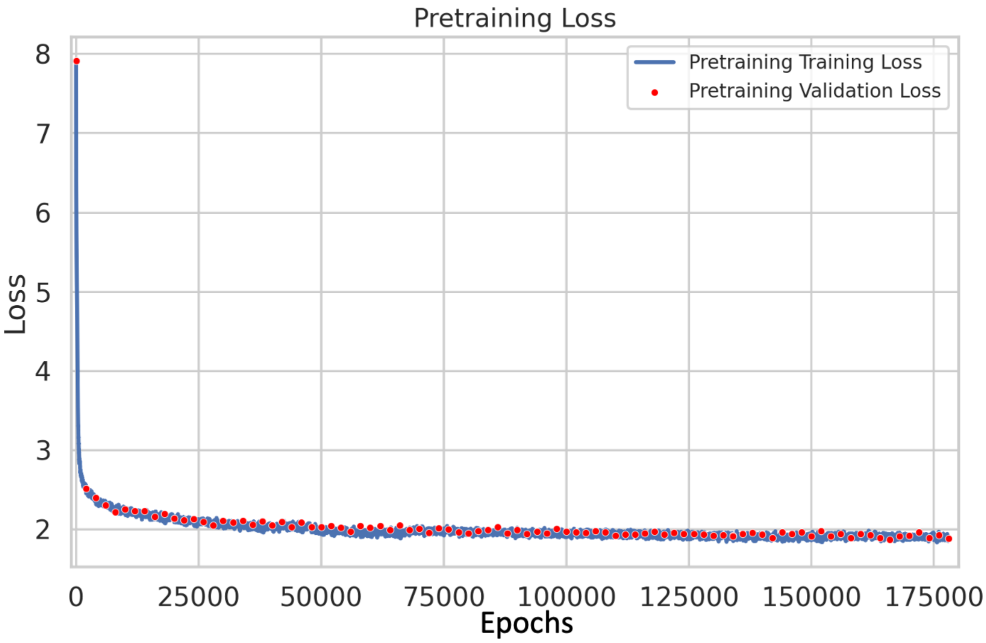

S1 Fig. Loss during pre-training

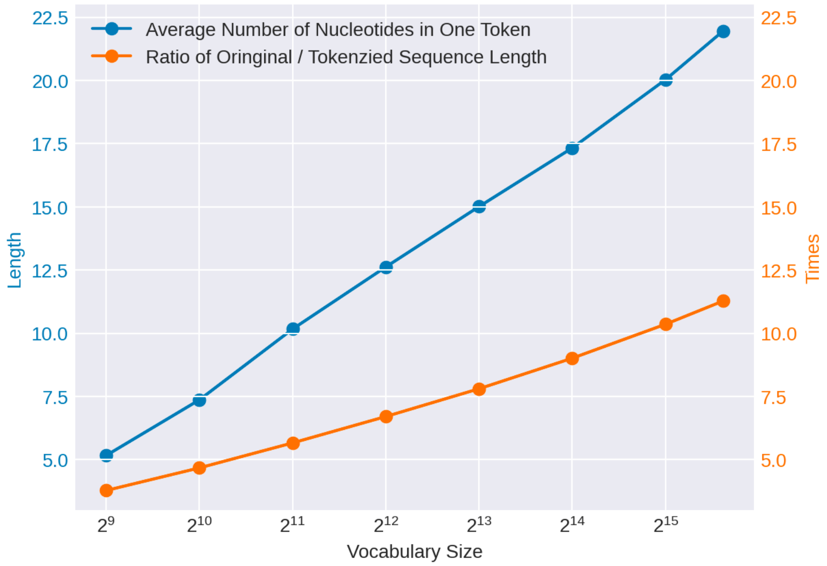

S2 Fig. Effect of Vocabulary Size on Token Length and Ratio of Original/ Tokenized Sequence Length

Our training dataset encompassed 11.6 billion nucleotides. A larger vocabulary size would reduce the number of tokens in the training data. Conversely, a smaller vocabulary size would restrict the length of sequences the model could process within the same context window.

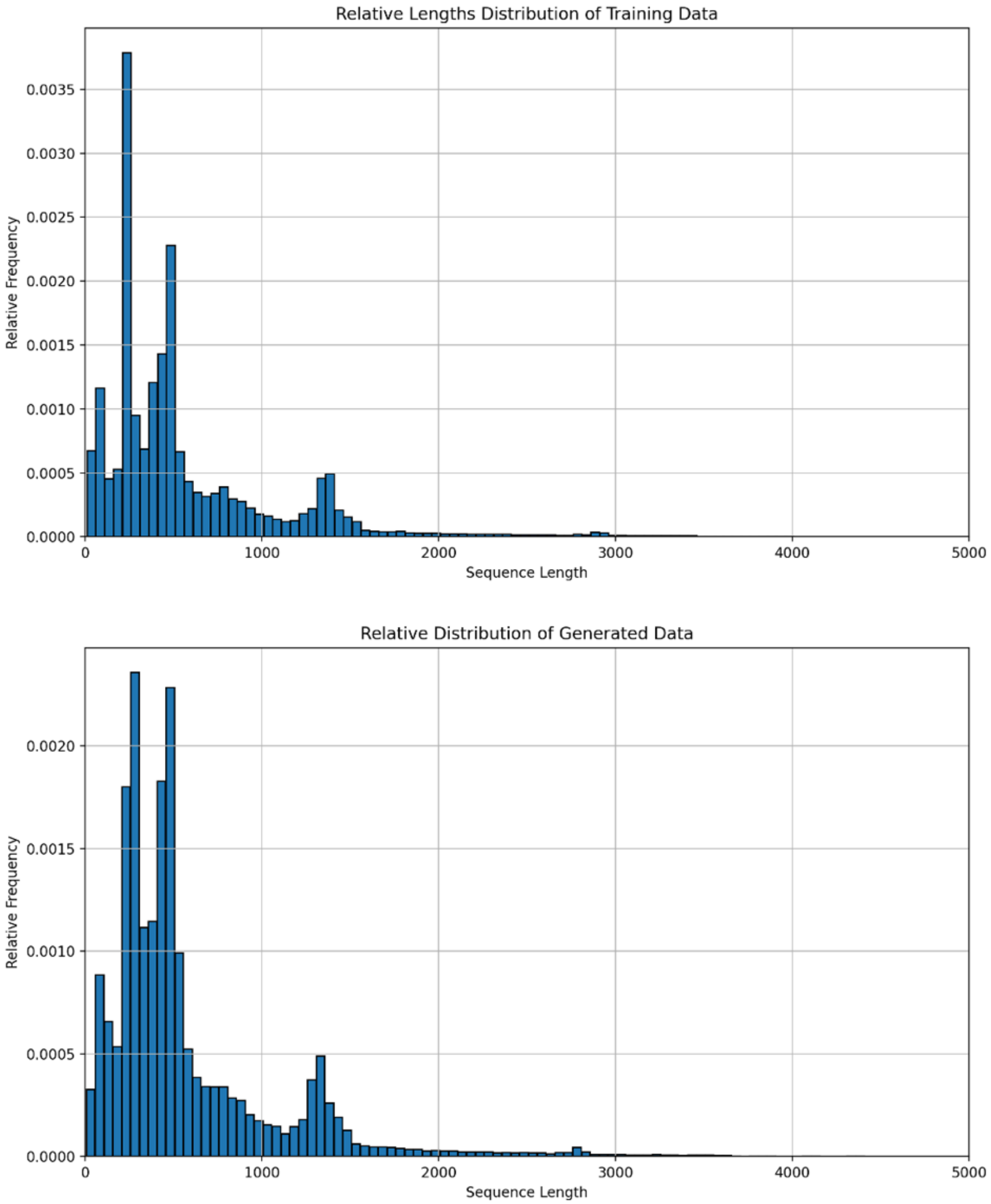

S3 Fig. Length distribution of generated sequences and sequences in the training dataset
The length distribution of the generated sequences is similar to that of the training dataset but slightly smoother.

S1 Table. Hyperparameters of *nhmmer* for homology search

	Query: RNA Generated by Pre-trained Model<br>DB: RNAcentral<br>(As mentioned in Section 2.5 of the manuscript,)	Query: RNA Generated by Fine-tuned Model<br>DB: Curated Protein-binding RNA<br>(As mentioned in Section 2.7&2.8 of the manuscript)	Remark
**-T**	**0**	**0**	Report target sequences with a bit score >= T
**--F3**	0.02 when sequence length < 50 nt	-	A heuristic acceleration parameter
**--watson**	True	False	Only search the top strand
**-Z**	Number of Sequence in RNAcentral	Number of Sequence of target DB	Size of the target database used for E-value calculation

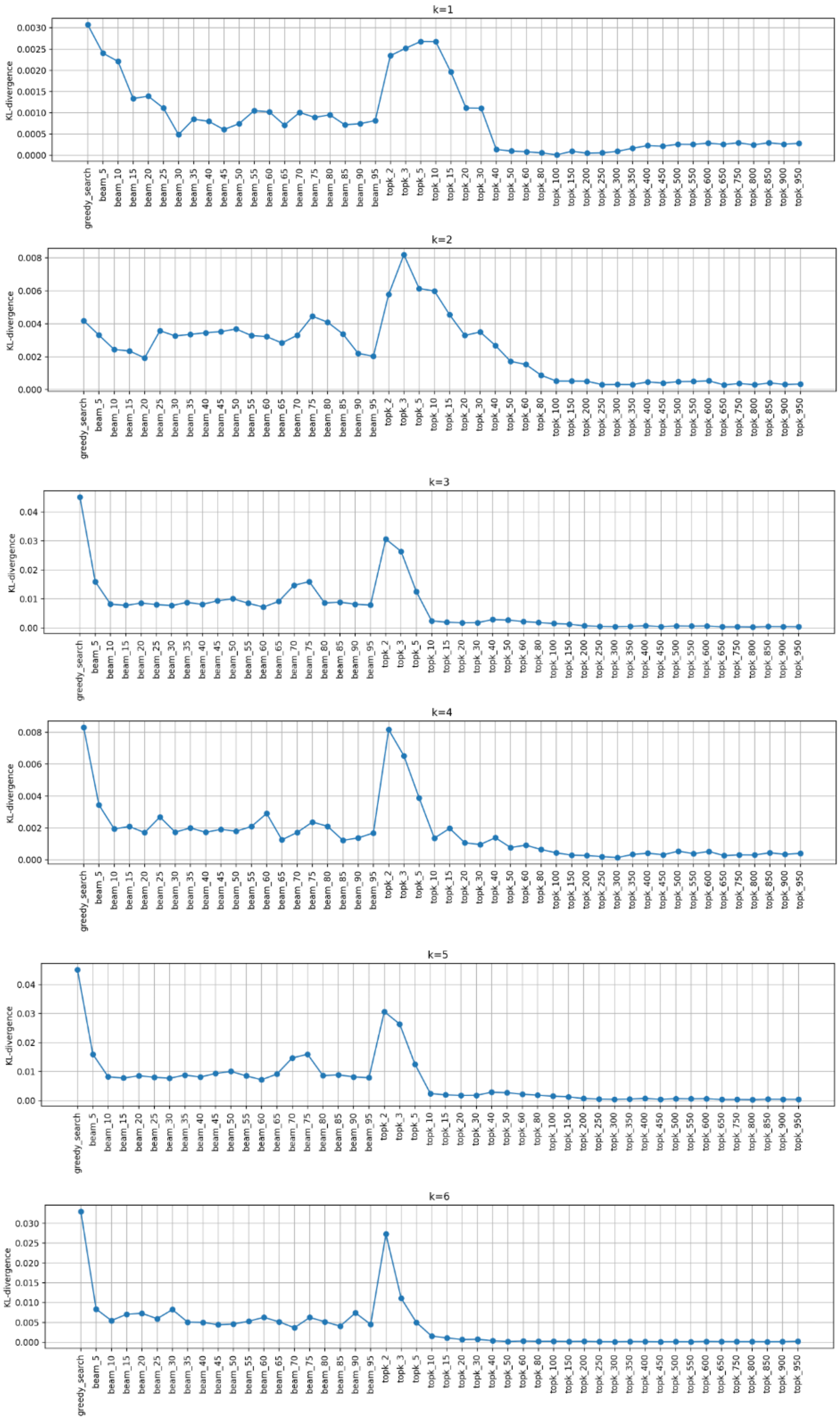

S4 Fig. KL-divergence of *k*-mer distributions between generated sequences under different sampling strategies and natural sequences at various *k*-mer lengths

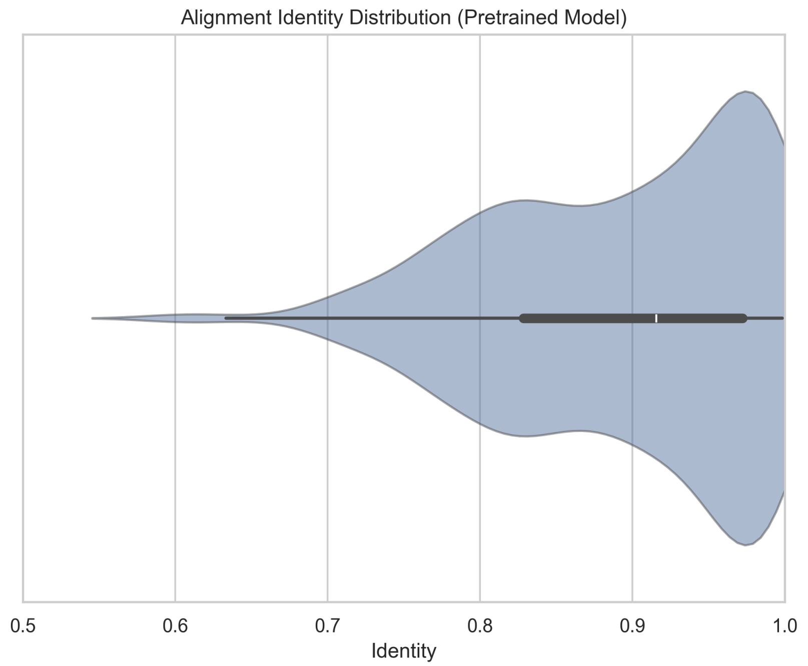

S5 Fig. Identity distribution of sequences generated by pre-trained model and aligned to any natural sequences.

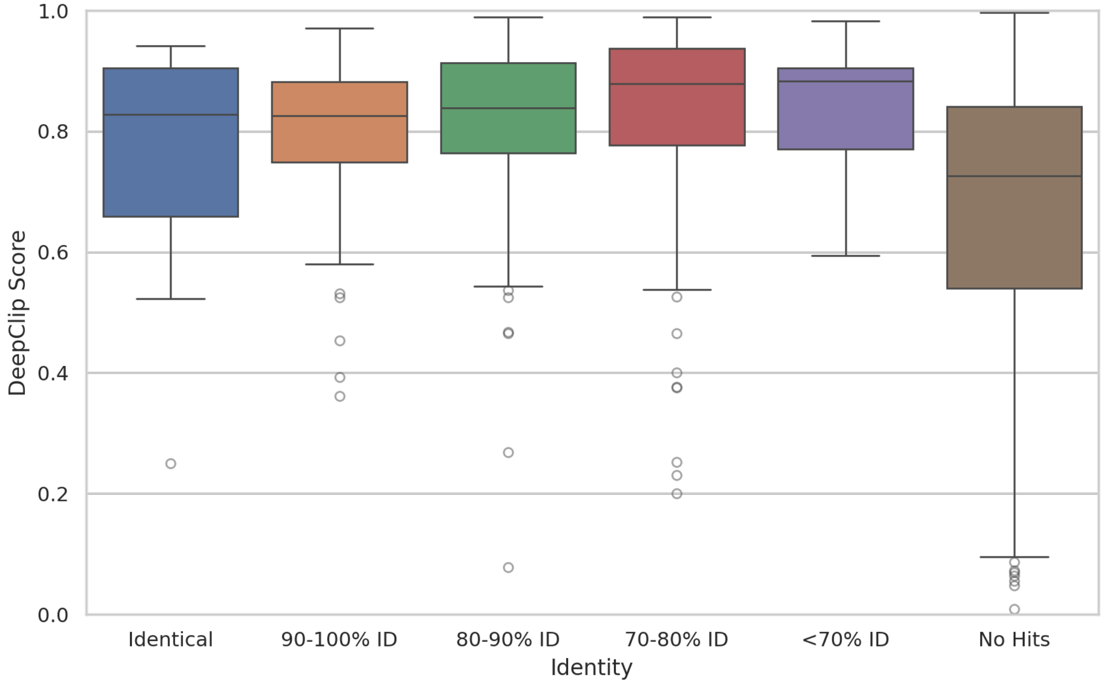

S6 Fig. Distribution of affinity scores with the target protein (SRSF1) at varying identity intervals.

In line with the experimental outcomes illustrated in Fig.6.b of the main text, the sequences generated by the fine-tuned GenerRNA encompass numerous RNA instances that, while exhibiting lower identity and, in some cases not aligned with any known sequences, nonetheless possess significantly elevated affinity scores.

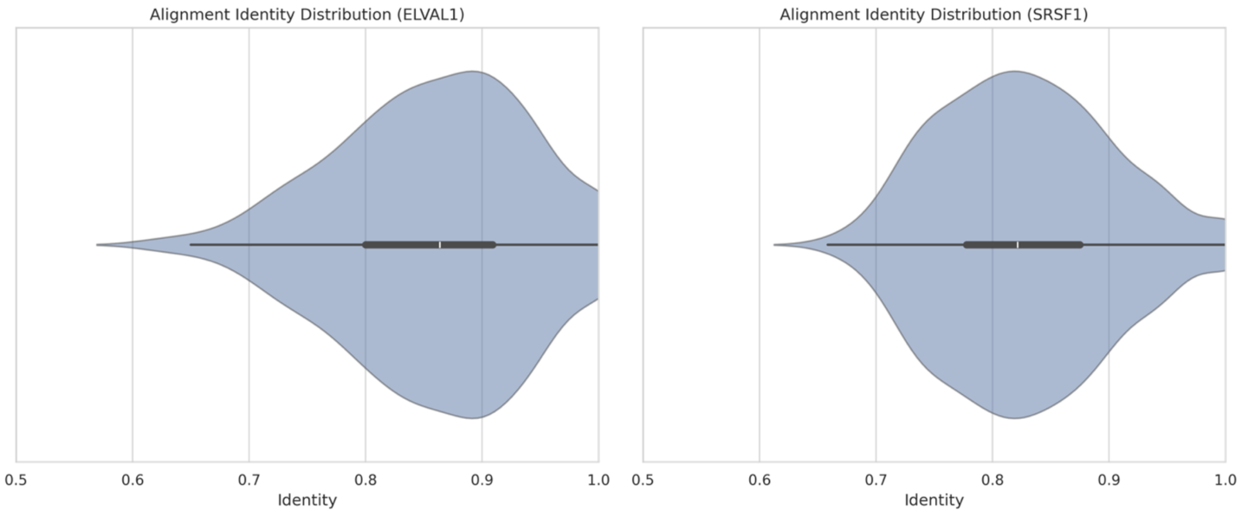

S7 Fig. Identity distribution of sequences generated by fine-tuned model and aligned to the training data

S1 Note. Detail about the comparative experiment

This section expands on Sections 2.7 and 3.4 regarding training RNAGEN on protein-binding RNA data. We observed that RNAGEN frequently encountered mode collapse or issues with critic loss and Wasserstein distance divergence in our dataset. After some preliminary adjustments on hyperparameters, we achieved relatively stable training over 100,000 epochs on both datasets by setting the learning rate to 3e-2, batch size to 64, and the gradient penalty to 5 while keeping the other training and model hyperparameters consistent with those of RNAGEN. Additionally, we limited the maximum sequence length accepted by the GAN model to 75 nucleotides, with shorter sequences padded to this length.

In the ELAVL1 dataset, RNAGEN effectively generated binding sequences, achieving an average affinity score of 0.773 (compared to GenerRNA's 0.872). Furthermore, only 57.9% of RNAGEN's sequences did not match any training data, whereas this proportion was 70.9% for GenerRNA. For sequences that aligned with known sequences, the identity distribution of RNAGEN was also slightly higher (one-tailed Mann-Whitney U test, $p = 0.0451$). However, in the case of the SRSF1 protein, while the average binding score of RNAGEN-generated sequences was significantly higher than that of background sequences (0.189), it only reached 0.439.

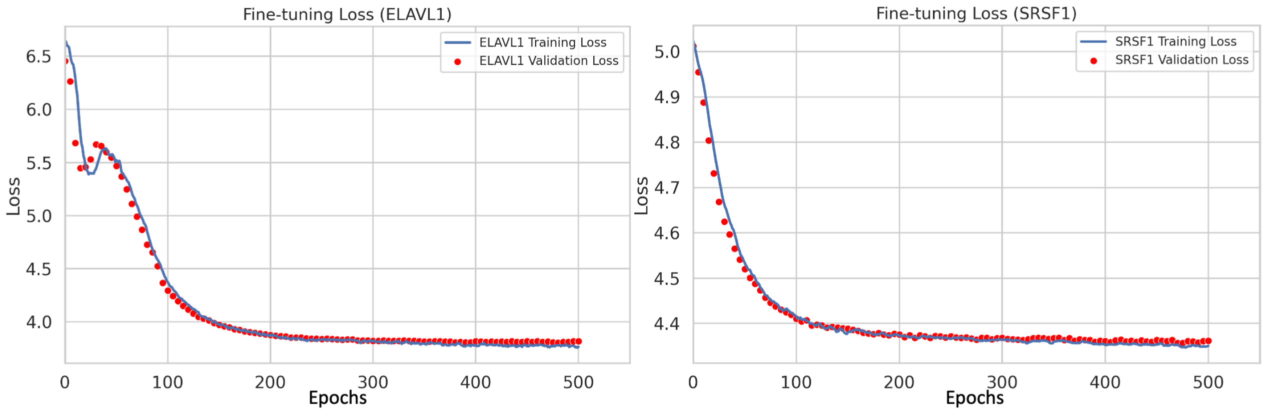

S8 Fig. loss during fine-tuning

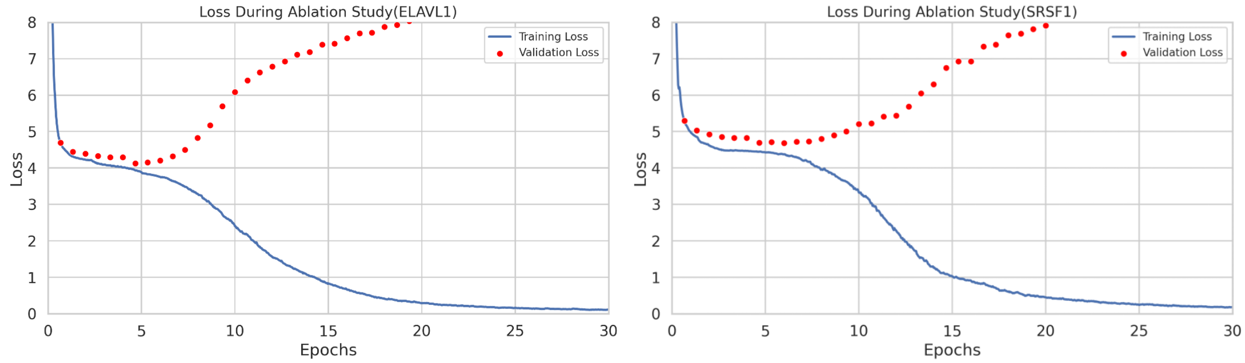

S9 Fig. Loss during ablation experiment

We chose the checkpoint with lowest validation loss for the generation.
